# Mutations in the alternative complement pathway in multiple myeloma patients with carfilzomib-induced thrombotic microangiopathy

**DOI:** 10.1038/s41408-023-00802-0

**Published:** 2023-02-27

**Authors:** Maria Moscvin, Christine Ivy Liacos, Tianzeng Chen, Foteini Theodorakakou, Despina Fotiou, Shahrier Hossain, Sean Rowell, Houry Leblebjian, Eileen Regan, Peter Czarnecki, Filippo Bagnoli, Niccolo’ Bolli, Paul Richardson, Helmut G. Rennke, Meletios A. Dimopoulos, Efstathios Kastritis, Giada Bianchi

**Affiliations:** 1 Amyloidosis Program, Division of Hematology, Brigham and Women’s Hospital, Boston, MA USA; 2 Stanford Health Care, Stanford, CA USA; 3 Department of Clinical Therapeutics, National Kapodistrian University of Athens, Athens, Greece; 4 Jerome Lipper Multiple Myeloma Center, Department of Medical Oncology, Dana-Farber Cancer Institute, Boston, MA USA; 5 Renal Division, Beth Israel Deaconess Medical Center, Boston, MA USA; 6 Department of Oncology and Onco-Hematology, University of Milan, Milan, Italy; 7 Hematology Division, Fondazione IRCCS Ca’ Grande Ospedale Maggiore Policlinico, Milan, Italy

**Keywords:** Cancer therapy, Haematological cancer

## Abstract

Thrombotic microangiopathy (TMA) has been reported to occur in multiple myeloma (MM) patients in association with treatment with carfilzomib, an irreversible proteasome inhibitor (PI). The hallmark of TMA is vascular endothelial damage leading to microangiopathic hemolytic anemia, platelet consumption, fibrin deposition and small-vessel thrombosis with resultant tissue ischemia. The molecular mechanisms underlying carfilzomib-associated TMA are not known. Germline mutations in the complement alternative pathway have been recently shown to portend increased risk for the development of atypical hemolytic uremic syndrome (aHUS) and TMA in the setting of allogeneic stem cell transplant in pediatric patients. We hypothesized that germline mutations in the complement alternative pathway may similarly predispose MM patients to carfilzomib-associated TMA. We identified 10 MM patients with a clinical diagnosis of TMA in the context of carfilzomib treatment and assessed for the presence of germline mutations in the complement alternative pathway. Ten, matched MM patients exposed to carfilzomib but without clinical TMA were used as negative controls. We identified a frequency of deletions in the complement Factor H genes 3 and 1 (delCFHR3-CFHR1) and genes 1 and 4 (delCFHR1-CFHR4) in MM patients with carfilzomib-associated TMA that was higher as compared to the general population and matched controls. Our data suggest that complement alternative pathway dysregulation may confer susceptibility to vascular endothelial injury in MM patients and predispose to development of carfilzomib-associated TMA. Larger, retrospective studies are needed to evaluate whether screening for complement mutations may be indicated to properly counsel patients about TMA risk with carfilzomib use.

## Introduction

Thrombotic microangiopathy (TMA) is a diverse spectrum of disorders unified by common clinical features. TMA syndromes are defined by the presence of microangiopathic hemolytic anemia (MAHA), thrombocytopenia and organ damage [1]. The organs most commonly involved are kidneys and central nervous system [1]. Endothelial injury leading to activation of the coagulation cascade, consumptive coagulopathy and small-vessel thrombosis with resultant tissue ischemia is the pathogenic driver of TMA syndromes [1].

TMA syndromes are classified in four groups based on their underlying etiology: (1) autoantibodies against ADAMTS13 causing thrombotic thrombocytopenic purpura (TTP); (2) infections with *E.Coli* O157 producing Shiga toxin leading to hemolytic uremic syndrome (HUS); (3) mutations in the complement regulatory pathways causing atypical HUS (aHUS); and (4) drug-mediated TMA [1]. Drug-mediated TMA is a rare but serious complication of treatment with numerous drugs including, but not limited to, quinine, gemcitabine, clopidogrel, VEGF-inhibitors, tacrolimus and cyclosporine. Drug-associated TMA can lead to severe renal failure requiring commencement of renal replacement therapy (RRT) [2].

Carfilzomib is an epoxyketone, irreversible proteasome inhibitor (PI) targeting the chymotrypsin-like activity [3, 4]. Carfilzomib is FDA approved in combination with lenalidomide and dexamethasone or daratumumab and dexamethasone for multiple myeloma (MM) patients who have received at least one prior line of therapy or as a doublet in combination with dexamethasone in patients who have previously received lenalidomide and bortezomib [5–8]. Initial registration studies of carfilzomib did not report TMA as an adverse event. However, clinically significant renal and cardiovascular complications presumably related to carfilzomib use have since been reported [9, 10]. Recently, a number of reports linking the use of PI to TMA have surfaced with carfilzomib being involved more frequently than the other two FDA-approved PIs, bortezomib and ixazomib. Importantly these two compounds are chemically distinct from carfilzomib, being boronic acid, and leading to reversible proteasome inhibition [11–16]. As the pathogenic mechanisms underpinning PI-related TMA remain unclear, there is no predictive biomarker to aid in patient risk-stratification and/or counseling. However, PI-related endothelial damage has been suggested as a potential driver of PI-related TMA [12, 17].

Germline genetic testing in TMA patients showed deregulation of complement genes located on chromosome 1q. Mutations in complement factor H (CFH), membrane cofactor protein (MCP), and CFHR5 genes have been reported in patients diagnosed with aHUS [18]. On this basis, the use of eculizumab, a humanized monoclonal antibody that stabilizes the terminal complement protein C5, has been approved for treatment of patients with aHUS [11, 14, 19]. Seminal work in the pediatric patient population undergoing allogeneic stem cell transplant identified germline deletions in the CFH-related genes 3 and 1 (delCFHR3-CFHR1) as risk factors for the development of TMA [20–23]. Defective complement regulation leads to uncontrolled, sustained activation of the alternative complement pathway resulting in endothelial injury and TMA.

We hypothesize that germline mutations in the complement alternative pathway are risk factors for development of TMA in carfilzomib-treated, MM patients. We identified 10 patients with relapsed refractory MM who developed TMA while on treatment with carfilzomib and 10 matched control MM patients who received similar cumulative dose and schedule of carfilzomib-based regimens but who did not develop TMA. We performed targeted DNA sequencing of peripheral blood mononuclear cells to identify germline mutations in the complement alternative pathway and compared the incidence of mutations between the two cohorts of patients and against the general population. Our data show that such mutations occur with a higher frequency in patients who develop TMA as compared to matched control, suggesting a potential causative role.

## Methods

### Patient population

We identified ten consecutive patients who developed TMA between 2005-2020, while receiving treatment with carfilzomib at the University of Athens in Greece or at the Dana Farber Cancer Institute in the United States. A clinical diagnosis of carfilzomib-induced TMA was made based on the fulfillment of the following criteria: active carfilzomib treatment; new onset of microangiopathic hemolytic anemia with decreased haptoglobin, thrombocytopenia and evidence of schistocytes on peripheral blood smear; oliguric, acute kidney injury network (AKI), AKIN ≥I (defined as a rise in serum creatinine relative to baseline of at least 1.5 times or 0.3 mg/dl and reduced urine output to less than 0.5 mL/kg/h within 48 h); in the absence of an acute infectious process, decreased ADAMTS13 activity and positive Coombs test. The control group included 10 patients treated with carfilzomib at the same institutions during 2015–2021 and who did not develop TMA. Controls were matched to the patient cohort based on age, gender, carfilzomib dose/duration and use/type of anti-platelet/anticoagulant agent. All subjects in the study were enrolled in IRB-approved protocols at the respective institutions. This study was conducted in accordance with the Declaration of Helsinki.

### Genetic renal sequencing

Complement pathway genetic testing was performed. For each patient and each control individual, genomic DNA was obtained from peripheral blood mononuclear and targeted sequencing was performed at the Molecular Otolaryngology and Renal Research Laboratories (MORL) at the University of Iowa, by using the Genetic Renal Panel v7 platform. This assay is designed to amplify a panel of complement mediated disease genes: Complement Factor H (CFH), Complement Factor I (CFI), Membrane Cofactor Protein (MCP), Complement Factor B (CFB), Complement factor H-related protein 5 (CFHR5), Complement component 3 (C3), thrombomodulin (THBD), diacylglycerol kinase epsilon (DGKE), plasminogen (PLG), ADAM Metallopeptidase with thrombospondin type 1 motif 13 (ADAMTS13), Metabolism of Cobalamin Associated C (MMACHC) and Glucose-6-phosphate dehydrogenase (G6PD) [24–27]. Deep intronic or regulatory region variants are not detected with this assay. Multiplex Ligation-Dependent Probe Amplification (MLPA) was performed to detect deletions delCFHR3-CFHR1 and delCFHR1-CFHR4 at the level of the regulator of complement activation (RCA) locus, on chromosome 1q32.2. The presence of both alleles was considered a normal test. The absence of one allele was reported as a heterozygous gene deletion, and the absence of both alleles was reported as a homozygous gene deletion.

## Results

We identified ten consecutive MM patients on active treatment with carfilzomib with clinical characteristics of TMA. Patients’ characteristics and laboratory values at the time of TMA diagnosis are reported in Tables 1, 2, 3 and Table s1. The median time to diagnosis of TMA, calculated from commencement of carfilzomib-based therapy, was 4.5 months (range 1–60 months). Clinical diagnosis was confirmed histologically by renal biopsy in three subjects (patients 3, 6 and 10).

**Table 1:** Patient characteristics and prior therapies.

Patient	Age (years)	Gender	Ig Isotype	ISS at diagnosis	FISH	Number of prior lines of therapy	PI before/after carfilzomib	Previous ASCT (years prior to diagnosis of TMA)
1	70	F	IgGκ	2	+1q21; t(4; 14); del17p	1	Ixa before/Bor after	N
2	69	F	IgGκ	2	N/A	2	Bor before	Y (3)
3	67	M	IgGλ	1	N/A	2	Bor before	Y (11)
4	51	M	IgGκ	3	N/A	1	Bor before	Y (1)
5	66	M	IgGκ	3	+1q21; del13q; del17p	1	Ixa before /Bor after	N
6	70	F	IgGλ	3	1q21	1	Bor before	Y (4)
7	55	M	IgGλ	1	N/A	5	Bor before	Y (12)
8	73	M	IgGλ	3	Normal	1	Bor before	Y (4)
9	73	F	IgGλ	3	+1q21	5	Bor before	N
10	47	M	IgGλ	2	t(11;14)	3	Bor before	Y (2)

The table shows age and gender of patients as well as Ig isotype, staging and FISH findings at time of diagnosis. Prior therapies, including previous and subsequent proteasome inhibitor use and stem cell transplant are reported.

*F* female, *M* male, *del* deletion, *Bor* bortezomib, *Ixa* ixazomib, *PI* proteasome inhibitor, *ASCT* Autologous stem cell transplant, *N* no history of ASCT, *Y* patient received ASCT.

**Table 2:** Clinical presentation of TMA.

Patient	Hgb (g/dL)	Platelet count (×10^9^/L)	Cr (mg/dL)	Baseline Cr (mg/dL)	LDH (U/L)	Anuria/Oliguria	GI sx	New HTN at time of TMA diagnosis	TMA confirmed by renal biopsy
1	7.7	30	2.0	0.65	619	Y	N	Y	N
2	7.7	20	3.08	1.19	371	Y	N	Y	N
3	13	31	1.04	0.86	240	N	N	N	Y
4	7.9	10	11.66	0.8	2150	N	Y	N	N
5	12.3	190	2.40	0.74	451	Y	N	N	N
6	9.7	139	1.42	0.82	262	Y	N	Y	Y
7	9.0	10	0.92	0.81	635	Y	N	N	N
8	9.9	223	2.05	1.38	141	Y	N	Y	N
9	6.9	16	6.54	2.4	401	N	Y	N	N
10	10.5	23	4.63	1.3	2150	Y	N	N	Y

The table shows pertinent laboratory values, signs and symptoms occurring at the time of TMA diagnosis. For specific patients, definitive histo-pathologic diagnosis of TMA was obtained through renal biopsy.

*Hgb* hemoglobin, *Cr* creatinine, *GI sx* gastrointestinal symptoms, *HTN* hypertension, *Y* yes, *N* no.

Normal range values: Hgb 14–17.5 g/dl (male) and 12–15.5 (female); Plt 140–400 × 10^9^/L, LDH normal range: 135–225 U/L.

**Table 3:** Drug regimens and clinical management of TMA.

Patient	K dosage/schedule	K regimen	Timing^a^	Drug intervention	Clinical outcome of TMA	AC at the time of event
1	56 mg/m^2^/ twice weekly	Kd	2 months	PLEX	Resolved	N
2	56 mg/m^2^/ twice weekly	DaraKd	5 months	PLEX	Resolved	N
3	56 mg/m^2^/ once weekly	Kd	5 years	N	Resolved	Heparin
4	56 mg/m^2^/ twice weekly	KRd	4 months	PLEX	Death	N
5	56 mg/m^2^/ twice weekly	Kd	2 years	N	Death	Acetylsalicylic acid
6	56 mg/m^2^/ twice weekly	Kd	4 months	N	Resolved	Acetylsalicylic acid
7	56 mg/m^2^/ twice weekly	Kd	1 months	PLEX	Resolved	Acetylsalicylic acid
8	56 mg/m^2^/ twice weekly	Kd	2 years	N	Resolved	Acetylsalicylic acid
9	70 mg/m^2^/ once weekly	Kd	1 months	PLEX	ESRD	Rivaroxaban
10	56 mg/m^2^/ once weekly^b^	KP	2 years	Eculizumab	ESRD^c^	Apixaban

Table shows carfilzomib dose used at time of diagnosis and the chemotherapy regimen. It is also reported the time from diagnosis, the management and the clinical outcome. Last column reports use of anticoagulation at the time of TMA diagnosis.

*K* carfilzomib, *Kd* carfilzomib/dexamethasone, *DaraKd* daratumumab/carfilzomib/dexamethasone, *PLEX* plasma exchange, *N* no, *ESRD* end stage renal disease.

^a^Timing indicates time between first dose of carfilzomib administered and TMA diagnosis.

^b^This patient was on maintenance K D1 and D15 and pomalidomide D1-21 at the time of diagnosis.

^c^Patient was dialysis dependent for 1 year before partially recovering renal function.

There was a male predominance (60%), with a median age of 68 years (47–73). Prior to carfilzomib, patients had received a median of 1.5 prior lines of therapy and seven patients (70%) had previously undergone autologous stem cell transplant 1 to 12 years prior to TMA diagnosis. Median laboratory values were as following: hemoglobin 9.4 g/dL (range: 6.9–13 g/dL), platelet count 26.5 × 10^9^/L (range 10–223 × 10^9^/L), creatinine 2.2 mg/dL (range 0.9–11.7 mg/dL), and LDH 426 U/L (range 141–2150 U/L).

Carfilzomib was discontinued at the time of diagnosis in all patients. All subjects were treated with supportive therapy, five patients received daily therapeutic plasma exchange (PLEX) for 5 sessions and one patient received eculizumab for a total of 6 doses. Upon discontinuation of carfilzomib, 6 out of 10 patients had resolution of TMA without need for renal replacement therapy. Two patients showed resolution of microangiopathic hemolytic anemia but required commencement of renal replacement therapy. Of these, one patient remains dialysis dependent today, while the other (patient #10) had partial recovery of renal function and was able to discontinue intermittent hemodialysis 9 months after the event with glomerular filtration rate stabilizing around 35 ml/min/m2. Two out of ten patients died as a consequence of progressive, multi-organ failure, involving both heart and kidneys.

**Fig. 1:**
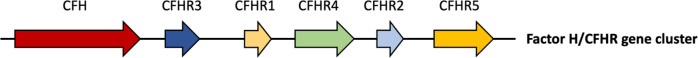
Haplotype block structure of CFH and CFH-related genes. The schema shows the region of complement activation gene cluster on chromosome 1q.

The results of germline genetic testing for completement mutations in TMA patients and controls are shown in Table 4. Deletions involving the CFHR3-CFHR5 region were detected in seven of the ten cases of TMA (70%), including 2 patients with homozygous gene deletion. Four patients had heterozygous deletion of CFHR3-CFHR1 and 1 patient had heterozygous deletion of CFHR1-CFHR4 (Fig. 1). Mutations in MCP (CD46) and CFHR5 were each identified in two of the TMA patients. Two patients had no detectable complement gene abnormalities, and one patient carried a CFHR5 mutation in the absence of any CFHR3-CFHR5 deletion.

**Table 4:** Complement pathway mutation screening and MLPA analysis.

Patient	Genetic renal panel	Zygosity of mutated gene(s)	MLPA	Zygosity of MLPA
Patients				
1	Normal	n/a	delCFHR3-CFHR1	Homozygous
2	CFHR5 (p.Met514Arg)	Heterozygous	delCFHR3-CFHR1	Heterozygous
3	Normal	n/a	Normal	n/a
4	MCP	Heterozygous	delCFHR1-CFHR4	Heterozygous
5	ADAMTS13 (p.Gly479 = ) MCP (p.Glu142Gln)	Heterozygous/ Heterozygous	delCFHR3-CFHR1	Homozygous
6	CFHR5 (p.Pro391Leu)	Heterozygous	Normal	n/a
7	Normal	n/a	delCFHR3-CFHR1	Heterozygous
8	Normal	n/a	Normal	n/a
9	Normal	n/a	delCFHR3-CFHR1	Heterozygous
10	n/a	n/a	delCFHR3-CFHR1	Heterozygous
Controls				
1	Normal	n/a	Normal	n/a
2	Normal	n/a	Normal	n/a
3	C3-VUS	Heterozygous	Normal	n/a
4	Normal	n/a	Normal	n/a
5	Normal	n/a	Normal	n/a
6	ADAMTS13-VUS	Heterozygous	delCFHR3-CFHR1	Heterozygous
7	Normal	n/a	delCFHR3-CFHR1	Heterozygous
8	Normal	n/a	Normal	n/a
9	Normal	n/a	Normal	n/a
10	THBD-VUS	n/a	delCFHR3-CFHR1	Heterozygous

Table shows genetic test results for both patients and controls.

*n/a* genetic abnormality not detected, *del* deletion, *CFHR* complement factor H related protein, *VUS* Variant of Unknown Significance.

The control patients had equal gender representation with a median age of 61 years (51–79). They received a median of 1.5 prior lines of therapy. All 10 control patients had normal laboratory values including hemoglobin, creatinine, LDH and no evidence of AKI while on treatment with carfilzomib. None of the patients in the control group had identifiable mutations in MCP, CFHR5, CFH, CFB, and CFI genes by direct gene sequencing. Heterozygous deletion of CFHR3-CFHR1 was detected in three control patients (30%). No homozygous deletions were observed in control patients.

### Illustrative case

Case #10 is a 49-year-old white man who was diagnosed with multiple myeloma (MM) in January 2017 when he presented with back pain. At diagnosis, he was found to have a T8 compression fracture and laboratory tests demonstrated hemoglobin 9.6 g/dL, platelet count 193.5 × 10^9^/L, LDH 139 U/L, creatinine 1.3 mg/dL, elevated total protein 12.6 g/dL, low albumin 2.5 g/dL and Beta 2 microglobulin 3.8 mg/L. Serum protein electrophoresis (SPEP) revealed a monoclonal IgG lambda paraprotein of 7.2 g/dL and bone marrow biopsy showed 40% lambda restricted plasma cells with presence of translocation t(11;14). Patient was diagnosed with International Staging System (ISS) stage 2, R-ISS 2 MM. He was started on induction therapy with lenalidomide/bortezomib/dexamethasone (RVD). After 1 cycle, 300 mg/m^2^ cyclophosphamide was added to intensify treatment. After 7 cycles of induction chemotherapy, the patient achieved a partial remission based on International Myeloma Working Group (IMWG) criteria. He underwent stem cell mobilization with G-CSF plus plerixafor and underwent an autologous stem cell transplant (ASCT) with standard melphalan 200 mg/m^2^ conditioning followed by uneventful infusion of 4.6 × 10^6^ CD34+ cells/kg. At restaging on day 90 post ASCT, patient was noted to have biochemical progression of MM with rising M spike. Carfilzomib/pomalidomide/dexamethasone (KPd) was started in November 2017. A baseline transthoracic echocardiogram (TTE) did not disclose any systolic or diastolic heart failure. For the first cycle, carfilzomib was administered at a dose of 20 mg/m2 intravenously (IV) on D1 and D2 and 27 mg/m^2^ on D8, D9, D15, and D16 of a Q28 day cycle. For subsequent cycles, carfilzomib was administered at 27 mg/m^2^ on D1/2, D8/9, and D15/16, with pomalidomide dosed at 3 mg by mouth (P.O.) D1-21 and dexamethasone at 20 mg P.O. D1/2, D8/9, D15/16, D22/23. Patient tolerated treatment well with no obvious side effect, except for insomnia and irritability, resolved with dose reduction of dexamethasone to 12 mg. Patient received 12 cycles of induction chemotherapy with KPd followed by commencement of maintenance with carfilzomib on D1/2 and D15/16 and pomalidomide D1-21 of a q28 day cycle. To ease schedule, patient elected to receive carfilzomib as a single weekly dose of 56 mg/m^2^ on D1 and D15 of a Q28 day cycle starting with cycle 15. His response at that time was consistent with a partial remission (PR), approaching a very good partial remission (VGPR). Of note, patient was therapeutically anticoagulated with apixaban at a 5 mg twice daily dose of as VTE prophylaxis in the setting of immunomodulatory drugs (IMiD)-based combinatorial treatment and prior history of deep venous thrombosis (DVT).

**Fig. 2:**
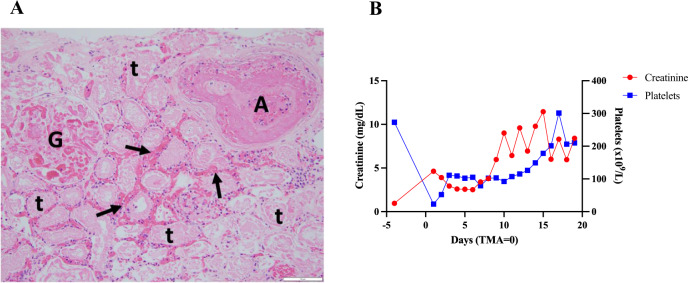
Illustrative histopathologic and laboratory findings in renal TMA. **A** Hematoxylin and eosin stain of a kidney biopsy obtained from TMA patient #10. Image shows extensive cortical necrosis, with recent thrombosis of a small interlobular artery (A), thrombosis of glomerular capillaries (G), congestion of peritubular capillaries and focal interstitial hemorrhage (arrows), and widespread tubular necrosis (t) and coagulative necrosis of all elements within the kidney cortex. **B** Trend of creatinine and platelet count for TMA patient #10. Days indicate hospital days, following TMA development.

Two months following this change in carfilzomib schedule, the patient presented to a local emergency department with new-onset dyspnea. He rapidly decompensated with acute cardiogenic shock complicated by hypoxemic respiratory failure and acute renal failure. On admission, TTE showed an acute drop in LVEF to 20% from 57% on a TTE obtained just 3 months prior as routine surveillance. He required endotracheal intubation and ventilatory support as well as vasopressor and inotropic support with norepinephrine, vasopressin and dobutamine. Workup revealed new onset of anemia (Hgb 10.5 g/dL), thrombocytopenia (platelets 23 × 10^9^/L) with high lactate dehydrogenase (LDH 2,150 U/L), low haptoglobin 29 mg/dL, and new onset AKIN III, anuric renal failure (creatinine 4.63 mg/dL from baseline 1.3 mg/dL). He was transferred to our center for further care. Schistocytes were present on peripheral blood smear (1+). An ADAMTS13 level was within normal limits. Testing for infections and direct antiglobulin test (DAT) Coombs were negative. He was started on continuous veno-venous hemofiltration (CVVH) on D1 after hospital transfer and subsequently transitioned to hemodialysis (HD) upon improvement of hemodynamics. A TTE on hospital day 3 showed resolution of acute left ventricular failure with an LVEF of 65%. Patient was extubated and vasopressor and inotropic support was discontinued on hospital day 4. However, patient remained anuric. Renal biopsy on hospital day 6 showed widespread cortical necrosis with only few areas of viability. All visualized vasculatures had thrombotic angiopathy findings consistent with TMA (Fig. 2A). He was diagnosed with atypical HUS, presumably related to carfilzomib. Upon multidisciplinary discussion, patient was commenced on intravenous eculizumab at a dose of 900 mg. He received his first dose on hospital day 8 and completed a total of 6 weekly doses before discontinuation. Eculizumab treatment was complicated by headache and hypertension. On hospital day 13, his platelet count began to uptrend (Fig. 2B). He was found to have a heterozygous CFHR1-CFHR4 deletion on germline testing. Patient was discharged in stable conditions on three times weekly hemodialysis nineteen days post hospital admission. He resumed pomalidomide plus dexamethasone (Pd) treatment upon discharge. Twenty-eight days after commencing hemodialysis, the patient started to produce small amount of urines. Urine output increased overtime alongside improved clearance. Patient was able to discontinue hemodialysis after 9 months from initiation. He was not re-exposed to PIs after this event. Unfortunately, the patient experienced relapsed/refractory multiple myeloma and eventually decided to enroll in hospice due to lack of meaningful therapeutic options. He passed away peacefully in July 2022.

## Discussion and conclusions

Several studies have described TMA in patients receiving carfilzomib [11–16]. The FDA Adverse Event Reporting System (FAERS) show a frequency of TMA after carfilzomib use of 0.97% of all reported adverse events, with 118 cases being serious events, including 30 deaths (1.31% of deaths for all causes). There are currently no biomarkers or predictive factors of carfilzomib-related TMA to aid in patient risk-stratification and counseling [28]. Prompt diagnosis and early intervention in drug-related TMA are challenging, as the laboratory features of mild TMA overlap with common MM and/or chemotherapy-related complications. In fact, if TMA is not suspected and appropriately worked up, anemia, thrombocytopenia, and renal failure can be erroneously attributed to underlying disease or chemotherapy.

There are a number of circumstantial evidences suggesting that complement-mediated endothelial damage may be at the base of carfilzomib-related TMA. Our group previously showed that pre-existent and/or acquired endothelial dysfunction as measured by non-invasive brachial artery flow-mediated dilatation (FMD) correlated with carfilzomib-related cardiovascular adverse events in a prospective study [29–31] A recent report showed complement activation by membrane attack complex (C5b-9) deposition on human endothelial cells exposed to plasma from patients during acute presentation of carfilzomib-mediated TMA [32]. A distinct study reported that PIs decrease CFH expression in glomerular epithelial cells in vitro, diminishing alternative complement pathway inhibition resulting in complement overactivation [33].

Studies carried out to investigate risk factors of TMA in the pediatric patient population undergoing autologous or allogeneic stem cell transplant identified a high frequency (83%) of heterozygous CFHR3-CFHR1 deletions, which are involved in complement overactivation and TMA development [23, 34]. The authors suggest that deletions in CFHR3-CFHR1 genes, despite being common in the general population, might influence recipients susceptibility to endothelial injury from high-dose chemotherapy or viral infections after HSCT [23].

In this proof-of-concept study, we detected a 2.3-fold increase in deletions in the CFHR3-CFHR1 region in carfilzomib-related TMA patients as compared to the general population, including 2 patients with homozygous deletions. In our control cohort, we observed deletions in CFHR3-CFHR5 in 30% of individuals, a frequency comparable to that of the general population, and solely in the heterozygous state [35]. Biallelic loss of CFHR3-CFHR1 genomic region induces uncontrolled complement activation and was found three times more frequently in aHUS patients as compared to the general population [36–39]. However, whether heterozygous deletion in CFHR3-CFHR1 represents a susceptibility genotype for endothelial damage-related disorders remains controversial as it has been reported as a protective factor for age-related macular degeneration but as a risk factor for systemic lupus erythematosus and aHUS [37, 38, 40].

Two patients in our TMA cohort harbored a single nucleotide mutation in the MCP gene (CD46) that had been previously reported in association with aHUS but whose functional significance is undetermined [41]. A novel single amino acid substitution in CFHR5 was detected in two distinct patients in our cohorts and its pathogenic potential is unknown [42, 43].

Importantly, 7 out of 10 patients in our cohort received carfilzomib at a dose of 56 mg/m^2^ twice weekly and one patient at a dose of 70 mg/m2 once weekly. A dose-effect relationship between carfilzomib and the occurrence of cardiovascular and renal has been previously reported, suggesting that higher peak doses may be associated with increased risk of endothelial damage.

Of note, two patients received bortezomib after occurrence of carfilzomib-related TMA without recurrence of diagnosis, suggesting that TMA may be the result of sustained endothelial damage in face of irreversible proteasomal inhibition.

To our knowledge, this is the largest series investigating genetic susceptibility in carfilzomib-related TMA.

Our study has several limitations. First, the sample size does not allow to define germline mutations in the complement inhibitory pathway as susceptibility alleles for carfilzomib-related TMA. However, we believe larger retrospective studies should be undertaken to ascertain the impact of these germline mutations on the risk of carfilzomib-related TMA in MM patients. Second, most of the patients in this study (7/10) received a doublet of carfilzomib/dexamethasone at a dose of carfilzomib of 56 mg/m^2^ twice weekly (for 6 patients) or 70 mg/m2 weekly (for one patient). The landscape of use of carfilzomib has substantially changed and carfilzomib in combination with IMiD is a frequently used regimen. Whether the presence of IMiD may impact incidence of TMA is not known, but conceivable. Third, only 3/10 patients in our cohort were receiving an anticoagulant at the time of the TMA diagnosis, albeit 4/10 were on acetylsalicylic acid. There were no patients concurrently receiving anticoagulation and an anti-platelet aggregation agent. Whether the prophylactic use of an anticoagulant or anticoagulant plus anti-platelet aggregation agent may ultimately mitigate the risk of developing TMA during treatment with carfilzomib remains unknown. Finally, patients in this study received variable TMA-directed treatment ranging from supportive care, through plasmapheresis as well as one patient treated with eculizumab. The small sample size does not allow to draw any conclusion regarding the merits of individual therapeutic interventions. As for other drug-related severe adverse events, prompt discontinuation of the offending drug and early administration of supportive therapy should be considered when there is high clinical suspicion of carfilzomib-induced TMA. Whenever safe and feasible, definitive histopathologic proof should be sought to aid in acute care and counseling regarding the future use of PIs.

In conclusion, our data suggest that in the context of carfilzomib-related endothelial damage, the presence of germline mutations in the complement inhibitory pathway may increase the risk of the development of TMA. It is likely that the development of TMA in the context of carfilzomib-related treatment is the result of several factors, including pre-existing endothelial damage and cardiovascular risk factors, biological variability in the kinetics of recovery of proteasome function in the endothelium after carfilzomib dosing, schedule and dosing of carfilzomib as well as genetic predisposition. Further studies are needed to fully elucidate the complex interplay of these genetic and environmental factors and to establish whether genetic testing for mutations in the complement inhibitory pathway may be helpful to better risk stratify and counsel MM patients about risk of TMA with carfilzomib treatment.

## Supplementary information

Table s1

## Data Availability

All data analyzed during this study are included in this published article and its supplementary information files.
