# Supplementary material for: Mutations in the alternative complement pathway in multiple myeloma patients with carfilzomib-induced thrombotic microangiopathy
Source: Blood Cancer J. 2023 Feb 27;13(1):31. doi: 10.1038/s41408-023-00802-0 (PMC9971259; doi:10.1038/s41408-023-00802-0)
Supplement: Supplementary file 1 — Table s1 [file 41408_2023_802_MOESM1_ESM.docx]

**Supplementary Table 1. Laboratory values.** Table shows laboratory values at baseline compared to values at diagnosis and highest value reached after diagnosis.

| **Patient** | **Hgb at diagnosis**  **(g/dL)** | **Min Hgb (g/dL)** | **Plt at diagnosis**  **(x10^9^/L)** | **Min plt (x10^9^/L)** | **Cr at diagnosis**  **(mg/dL)** | **Baseline Cr (mg/dL)** | **Max Cr (mg/dL)** | **LDH at diagnosis**  **(U/L)** | **Highest LDH**  **(U/L)** |
| --- | --- | --- | --- | --- | --- | --- | --- | --- | --- |
| 1 | 7.7 | 7.7 | 30 | 13 | 2.0 | 0.65 | 2 | 619 | 619 |
| 2 | 7.7 | 7.7 | 20 | 20 | 3.08 | 1.19 | 3.71 | 371 | 371 |
| 3 | 13 | 11.6 | 31 | 31 | 1.04 | 0.86 | 1.24 | 240 | 337 |
| 4 | 7.9 | 6.7 | 10 | 10 | 11.66 | 0.8 | 12.54 | 2150 | 2150 |
| 5 | 12.3 | 11 | 190 | 96 | 2.40 | 0.74 | 2.9 | 451 | 551 |
| 6 | 9.7 | 9.3 | 139 | 11 | 1.42 | 0.82 | 1.48 | 262 | 459 |
| 7 | 9.0 | 7.5 | 10 | 10 | 0.92 | 0.81 | 0.92 | 635 | 685 |
| 8 | 9.9 | 9.1 | 223 | 49 | 2.05 | 1.38 | 2.05 | 141 | 381 |
| 9 | 6.9 | 5 | 16 | 16 | 6.54 | 2.4 | 14.1 | 401 | 1181 |
| 10 | 10.5 | 10.5 | 23 | 23 | 4.63 | 1.3 | 4.63 | 2150 | 2150 |

Hgb, hemoglobin; Min, minimal value; Max, maximal value; Plt, platelets, Cr, creatinine.
